# Supplementary material for: Cross-species analysis of viral nucleic acid interacting proteins identifies TAOKs as innate immune regulators
Source: Nat Commun. 2021 Dec 1;12:7009. doi: 10.1038/s41467-021-27192-w (PMC8636641; doi:10.1038/s41467-021-27192-w)
Supplement: Supplementary file 1 — Supplementary Information [file 41467_2021_27192_MOESM1_ESM.pdf]

Supplementary Figure 1

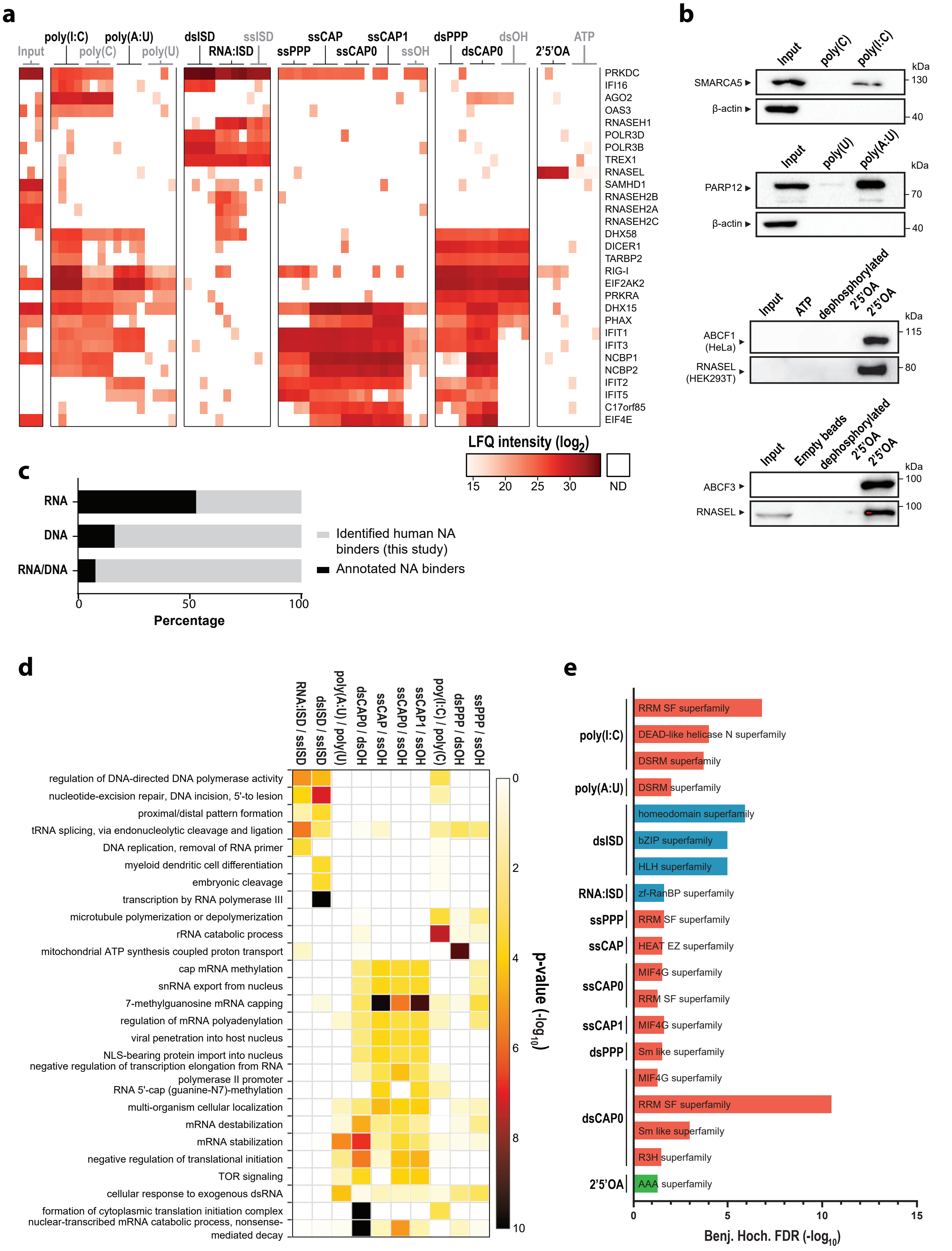

Supplementary Figure 1: AP-MS validation and enrichment analyses

(a) Log_2 LFQ intensities for a selected set of known nucleic acid binders identified in the THP-1 AP-MS screen. ND: not detected. Protein clustering is based on Euclidian distance and Ward as agglomeration method. **(b)** Western blots confirming nucleic acid binding for a selected set of AP-MS screen hits. Whole cell lysates (lysates from THP-1 cells were used, except for the ABCF1 and RNase L panel, where HeLa and HEK293T were used, respectively) were incubated with beads loaded with the indicated nucleic acid and co-precipitation of proteins was evaluated by western blotting against the indicated proteins. Data is representative of at least two biological repeats. **(c)** Percentage of proteins identified as interactors of RNA baits, DNA baits, and RNA/DNA baits that are already known NA binding proteins. **(d)** Gene Ontology Biological Processes (GOBP) terms enriched among the NA-binding proteins. The color denotes the $-\log_{10}$(p-value) (one-sided, unadjusted) of the Fisher Exact Test for the enrichment of the given GOBP term with the given NA bait. **(e)** Enrichment of known nucleic acid biding domains in the indicated AP-MS dataset. Domains with a Benjamini-Hochberg-corrected FDR < 0.05 were considered as significantly enriched (Fisher Exact Test) and RNA baits are colored in red, DNA baits in blue and 2'5'OA in green. Source data are provided as a Source Data file.

Supplementary Figure 2

a

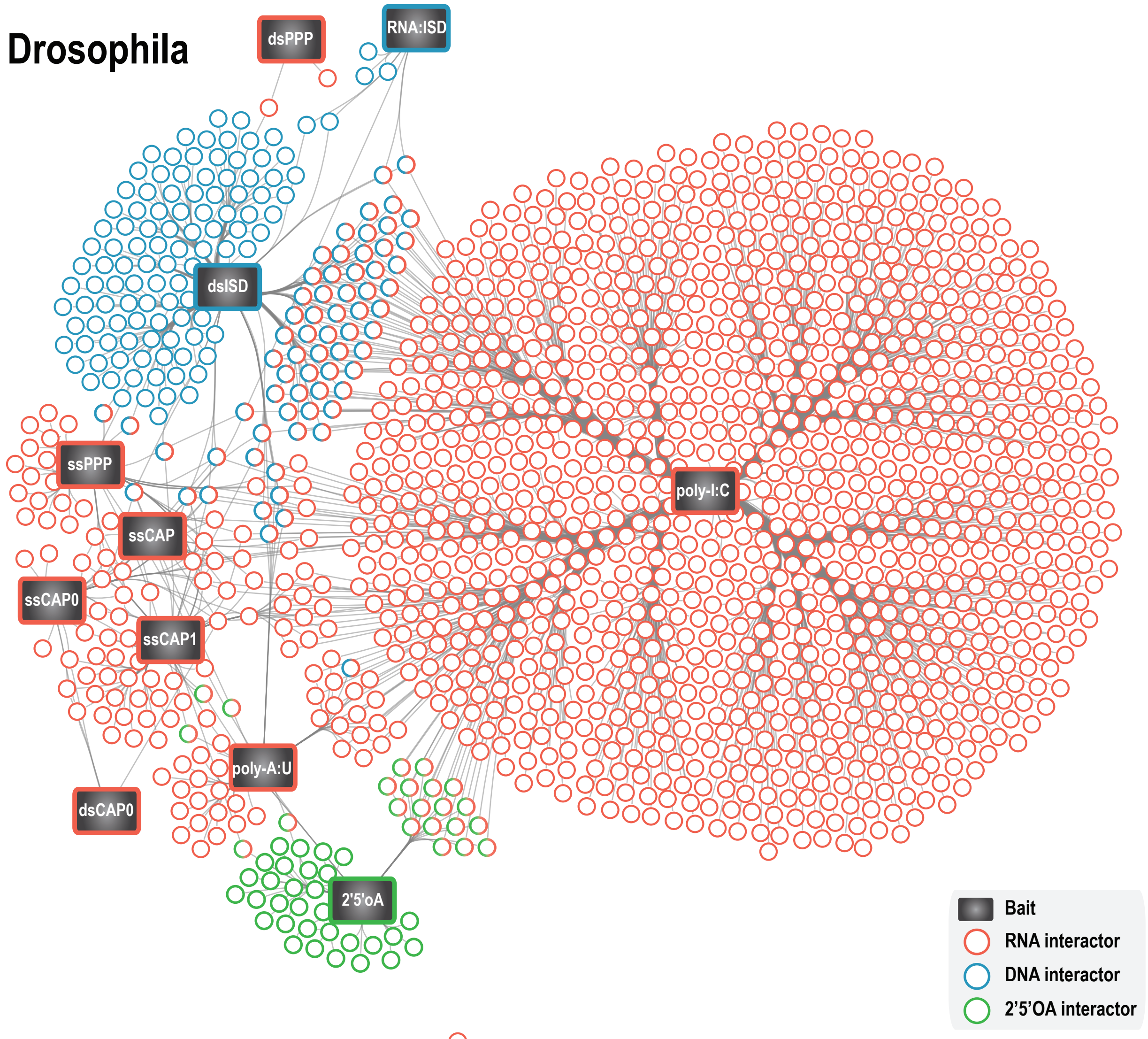

b

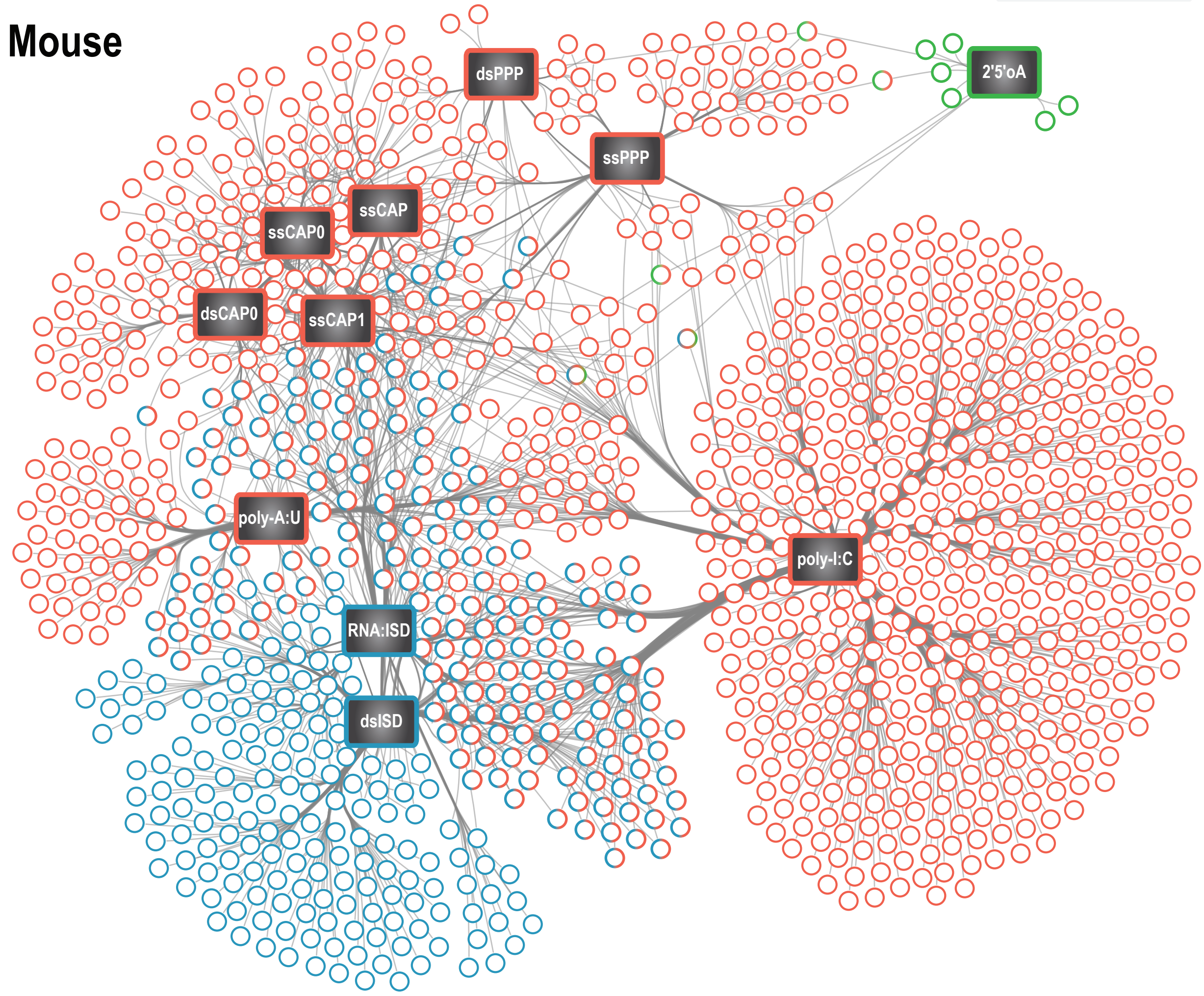

Supplementary Figure 2: Network analysis of fly and mouse AP-MS data

Network analysis of proteins identified to be significantly enriched in precipitates of the indicated NA bait (red: RNA baits, blue: DNA baits, green: 2'5'OA) in *drosophila melanogaster* (fly) **(a)** and mouse **(b)**. Significance was calculated using the Welch's t-test with an FDR < 0.05 (for the whole fly poly(I:C) samples the FDR was reduced < 0.001).

Supplementary Figure 3

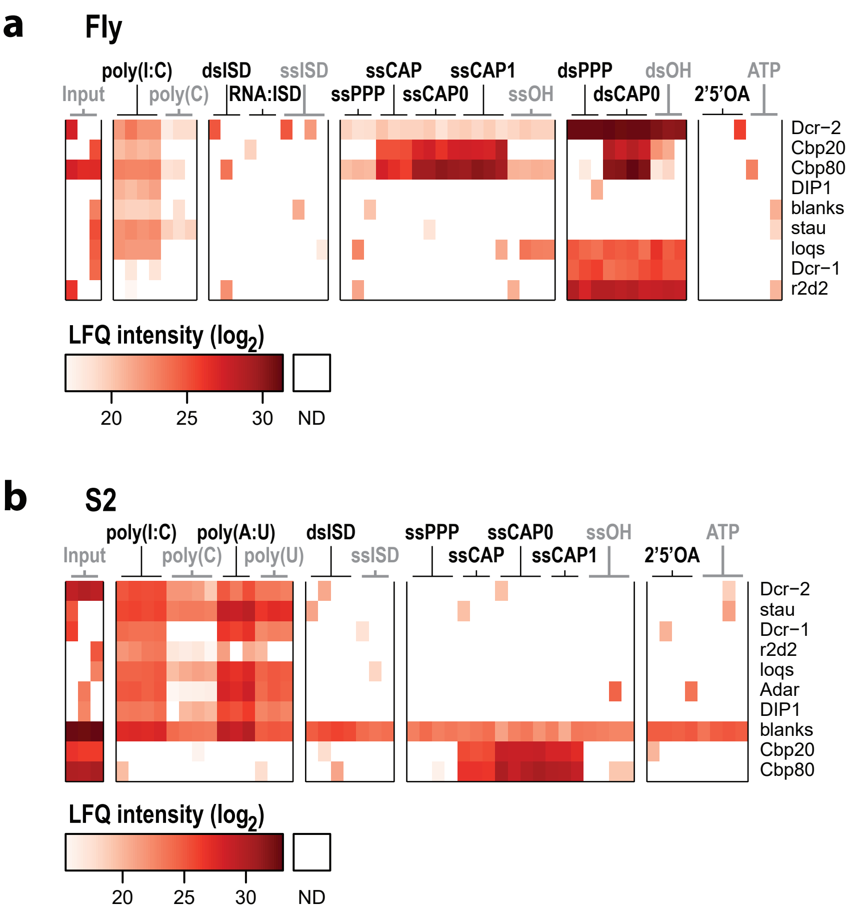

Supplementary Figure 3: Known NA binders identified in Fly and S2 cell lysates

Heatmaps displaying the log_2 LFQ intensity for the selected known NA interactors identified in fly **(a)** and Schneider S2 cells **(b)**. ND: not detected. Protein clustering is based on Euclidian distance and Ward as agglomeration method.

Supplementary Figure 4

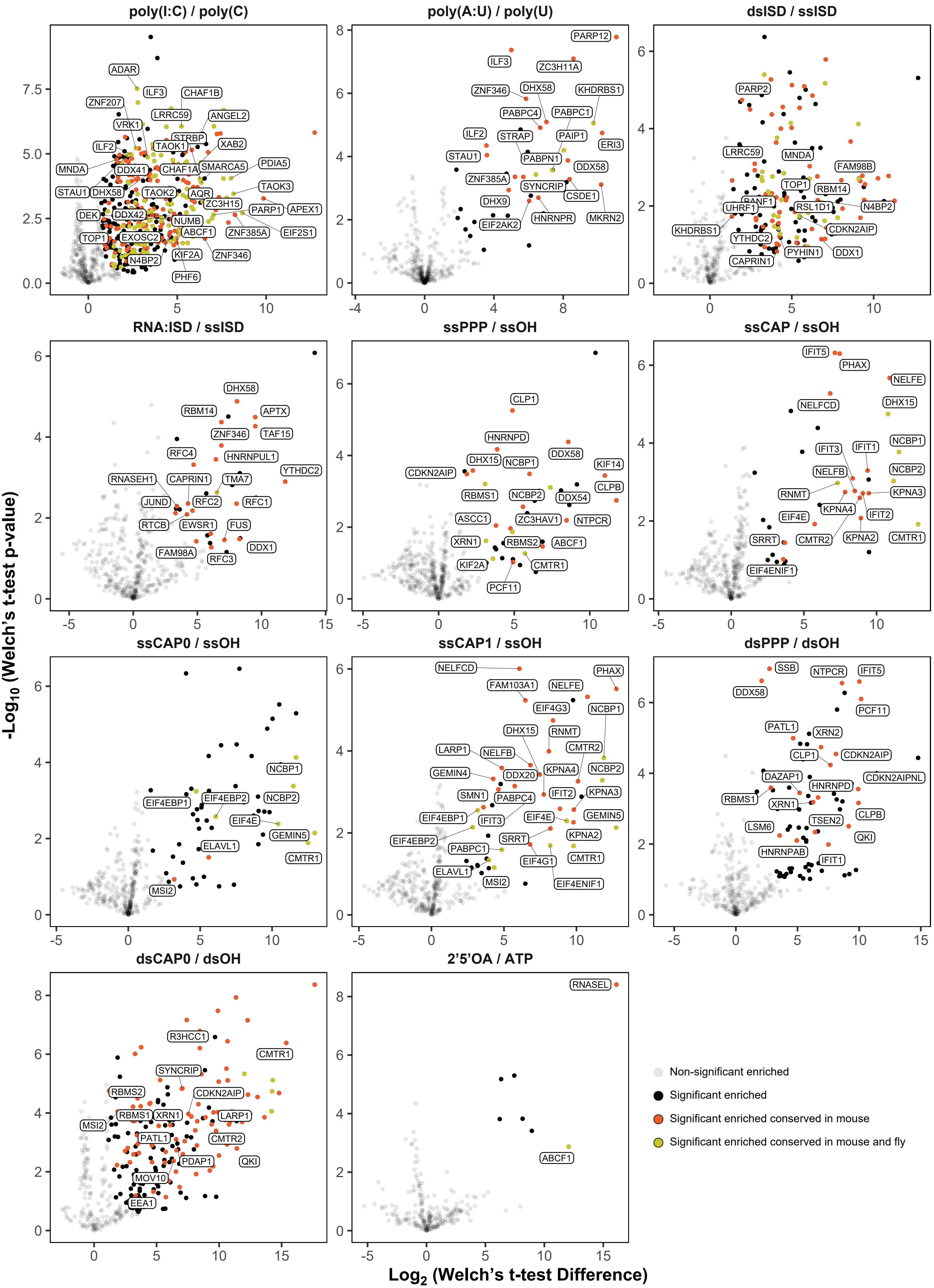

Supplementary Figure 4: Integrating binding information in human AP-MS screening

Volcano plots showing the $\log_2$ fold enrichment (*x*-axis) and $-\log_{10}$ p-value (*y*-axis) of significantly enriched proteins (black: significantly enriched, grey: non-significantly enriched) per bait/control comparison for the human AP-MS screening and considering conservation of this interaction in other baits. All enriched proteins with conserved binding patterns in mouse (red) or mouse and fly (yellow) are labelled, except for poly(I:C), dsISD and dsCAP0, where only a subset of candidates are labelled. Significance was assessed by a two-sided Welch's t-test (S0 = 1; min. 2 valid values in at least one group) using a permutation-based FDR of ≤ 0.05.

Supplementary Figure 5

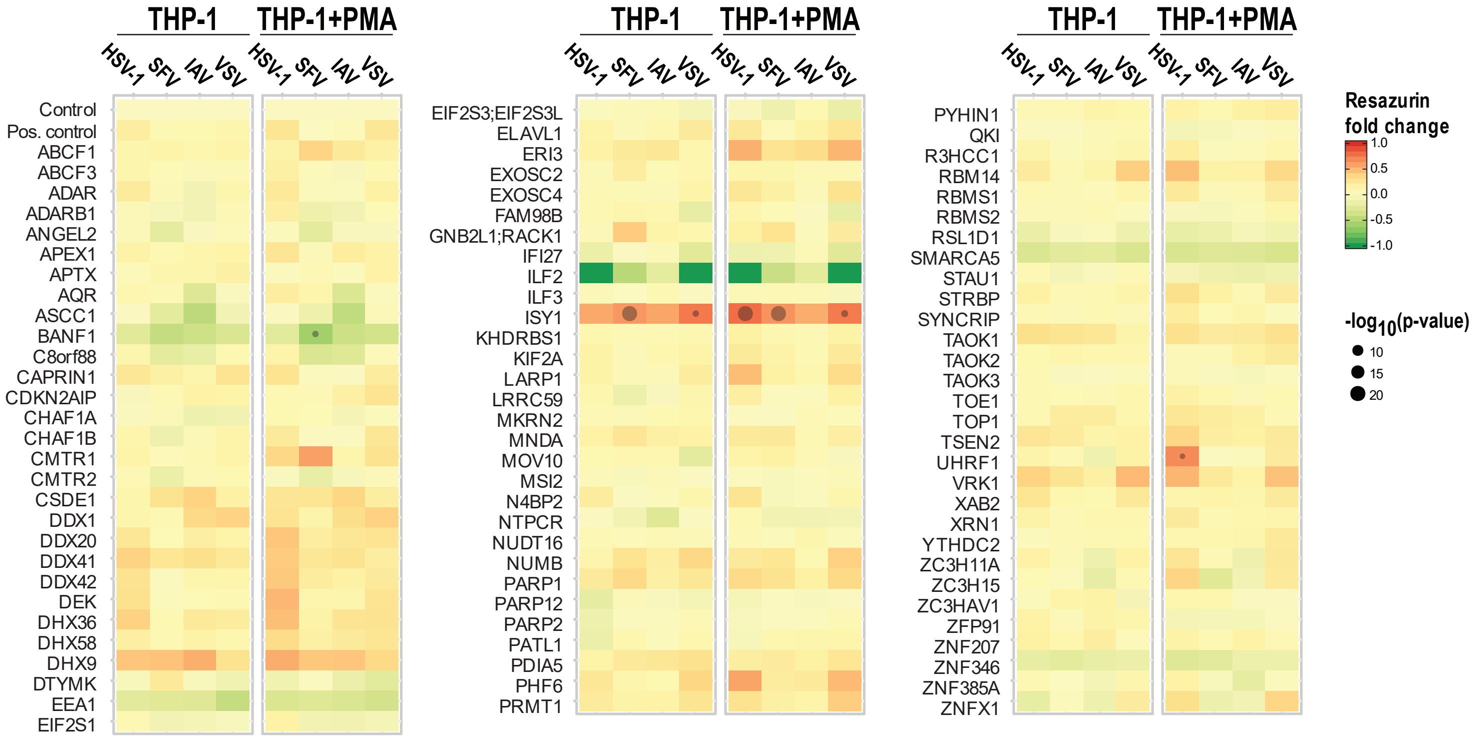

Supplementary Figure 5: Cell viability of infected THP-1 KO cells

Cell viability of THP-1 KO cells that were infected with the indicated viruses for 17 h. The screening was performed on THP-1 cells with or without PMA differentiation, as indicated. The color indicates the mean relative cell viability of three repeats as compared to the non-targeted control. The two-sided P-value is defined as probability that $\log_2(Luc_{KO}/Luc_C)$ is different from 0 using a random effects generalized linear Bayesian model; significant changes (p-value ≤ 0.05, unadjusted for multiple hypothesis testing; $Luc_{KO}/Luc_C \geq \log_2(1.5)$) are highlighted with dots. Data represents the median of biological triplicates. Source data are provided as a Source Data file.

Supplementary Figure 6

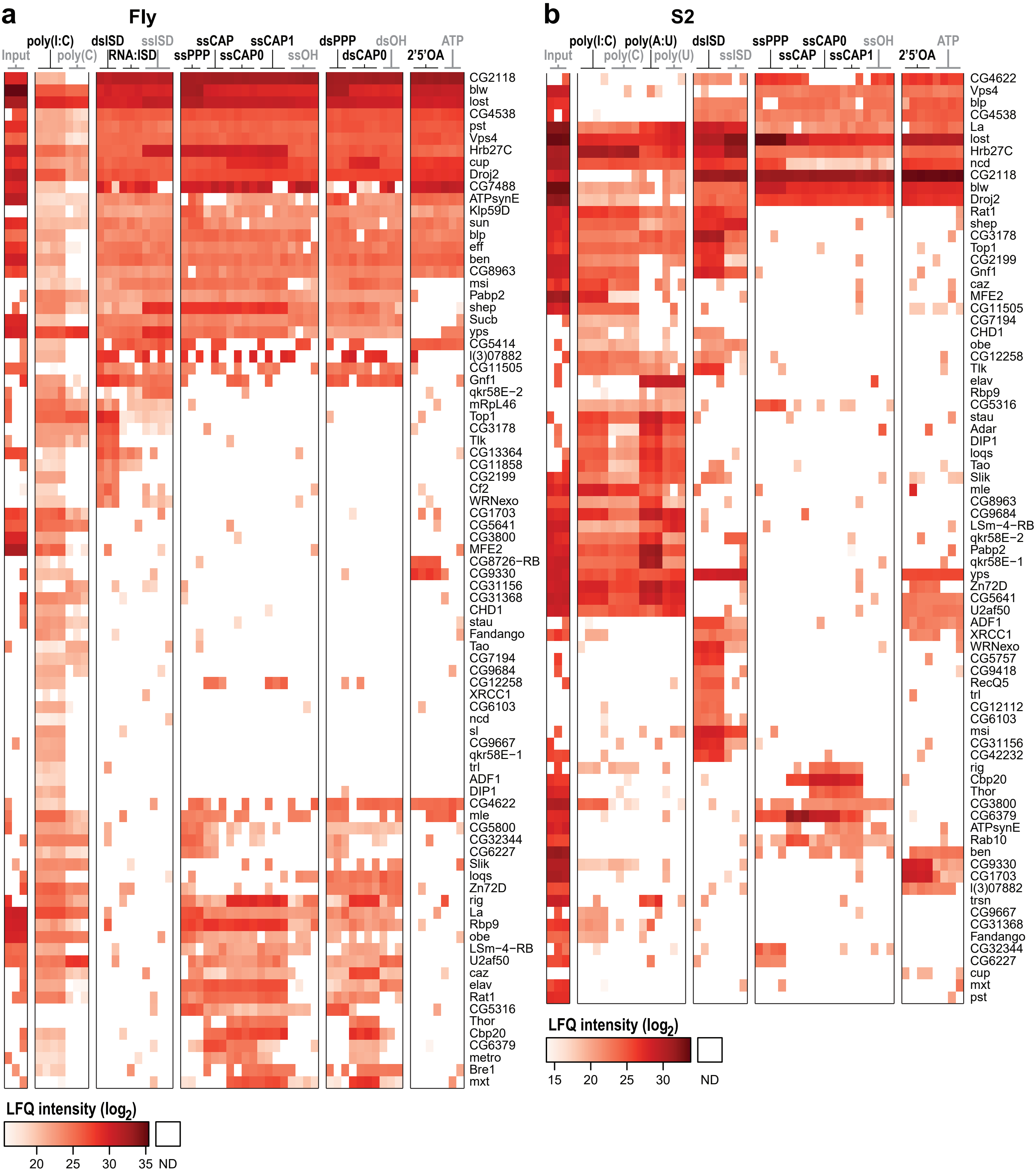

Supplementary Figure 6: Selected candidates identified in Fly and S2 cell lysates

Heatmaps displaying the $\log_2$ LFQ intensity for the selected candidates identified in fly **(a)** and Schneider S2 cells **(b)**. ND: not detected. Candidate clustering is based on Euclidian distance and Ward as agglomeration method.

Supplementary Figure 7

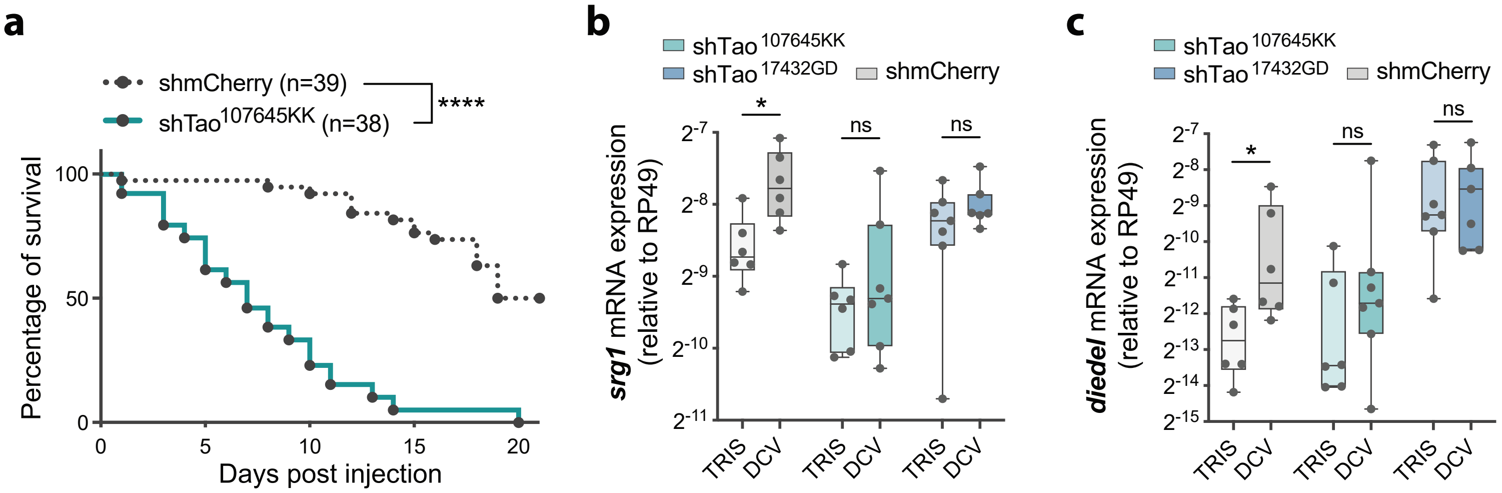

Supplementary Figure 7: Drosophila Tao silencing and its effect on gene expression

(a) Survival of Tao knockdown flies. Silencing of Tao expression in transgenic flies expressing a RNAi line targeting Tao (107645KK, green) using the Gal4-UAS system and the broadly expressed actin-Gal4 driver controlled by the thermosensitive (TS) tub-Gal80 repressor. shRNA targeting mCherry (black) was used as a control. Tao-depleted flies injected with TRIS buffer succumbed rapidly in comparison to control flies (shmCherry). **** $p < 0.0001$ as assessed by Log-rank (Mantel-Cox) test. **(b, c)** Expression of *Sting* (*srg-1*) and *diedel* mRNA in Tao depleted flies. Two independent RNAi lines were used to silence dTao (green: shTao 107645KK, blue: shTao 17432GD, grey: shmCherry control). Flies were infected via injection of DCV (500 pfu/fly) and gene expression was monitored by RT-qPCR at 2 days post infection. The plot shows data obtained from individual flies (dots). Center line, median; box limits, from the 25th to 75th percentiles; whiskers, from min to max. Statistical analysis was performed comparing the different lines and all the marker genes by Kruskal-Wallis test ($p = 1.37 \times 10^{-25}$, one-sided) with a Holm correction for multiple comparisons using the Dunn test (* $p < 0.05$). Source data are provided as a Source Data file.

Supplementary Figure 8

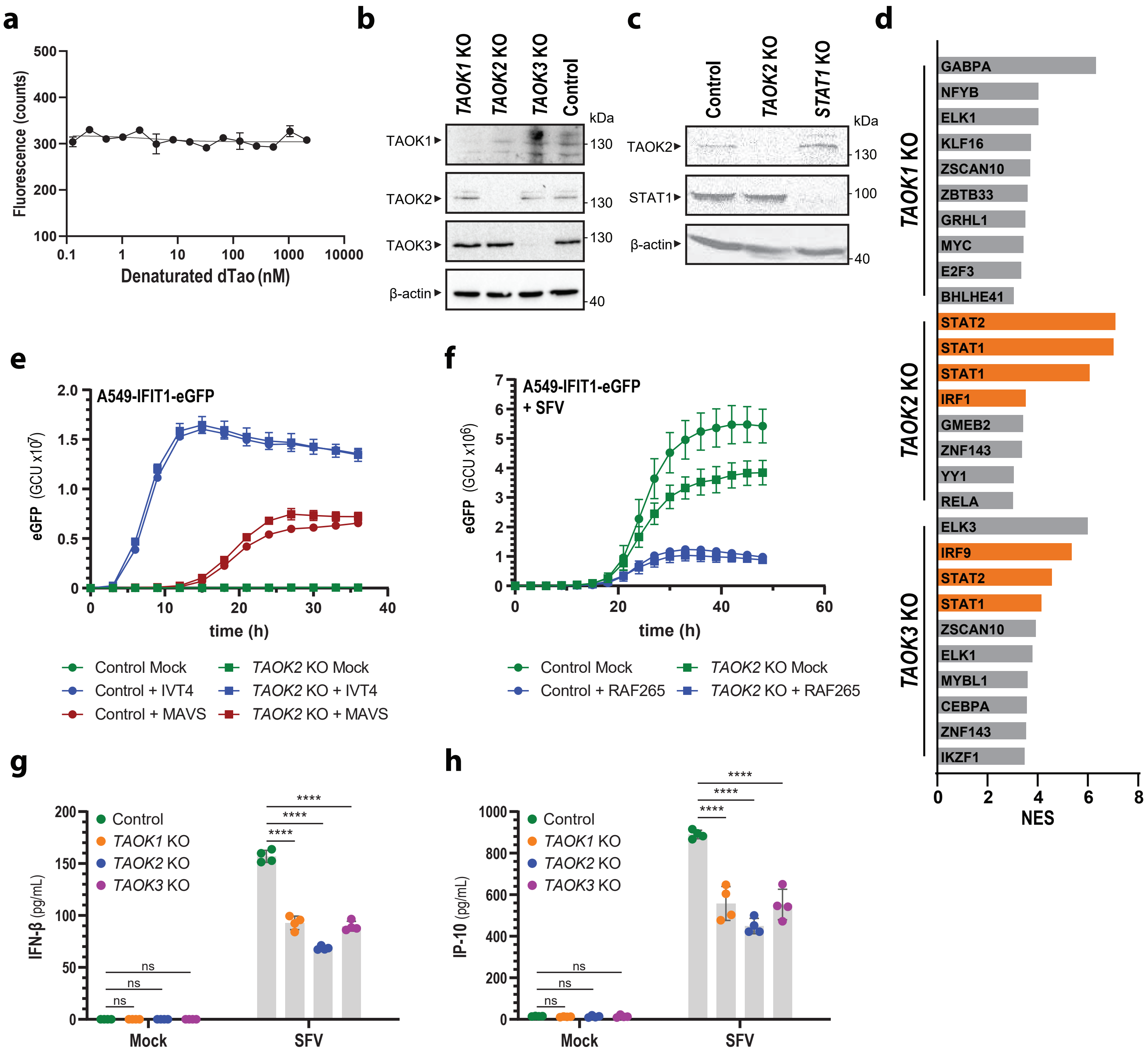

Supplementary Figure 8: TAOK supplementary data

(a) Fluorescent quenching assay testing the affinity between denatured dTao and poly(I:C). dTao was denatured by 1:1 dilution in 4% SDS and boiled at 95°C for 5 minutes followed by the fluorescence intensity measurement. Shown are mean fluorescence intensity (± SD) of three measurements. **(b)** Western Blot confirming KO of TAO kinases in THP-1 cells. One representative blot of two independent experiments is shown. **(c)** Western Blot confirming KO of *TAOK2* in A549-IFIT1-eGFP cells. One representative blot of one independent experiment is shown. **(d)** Upstream promoter analysis of proteins where a lack of upregulation is observed in SFV infected TAOK KO cells vs infected control THP-1 cells. Transcription factors linked to interferon-regulated innate immunity, based on Reactome pathway enrichment analysis, are marked in orange. Normalized Enrichment Score (NES) indicates the enrichment score of transcription factor. **(e, f)** Scrambled sgRNA control (circle) or *TAOK2* KO A549-IFIT1-eGFP cells (square) were transfected with IVT4 (100 ng/mL, blue), 100 ng pTO-SII-HA-MAVS expression plasmid (red) or PBS (green) **(e)** or were infected with SFV-mCherry (MOI 5) and simultaneously treated with the TAOK2 inhibitor RAF265 (500 nM, blue) **(f)**. Green fluorescence intensity was measured at the indicated time points using an IncuCyte S3 live cell imaging system. Mean green intensity per image (GCU) ± SD (*y*-axis) is shown over time (*x*-axis). Data presented in (e) is averaged across three and data in (f) is averaged across five biological replicates. **(g, h)** *TAOK1* KO (orange), *TAOK2* KO (blue), *TAOK3* KO (purple) or control (green) THP-1 cells were infected with SFV (MOI 1) and 24 h later the accumulation of IFN-β **(g)** and IP-10 **(h)** in the supernatant was measured by ELISA. Data presented is averaged across four biological repeats ± SD, **** $p < 0.0001$ (Two-way ANOVA with Šídák's multiple comparison test). ns: not significant. Source data are provided as a Source Data file.

Supplementary Figure 9

a

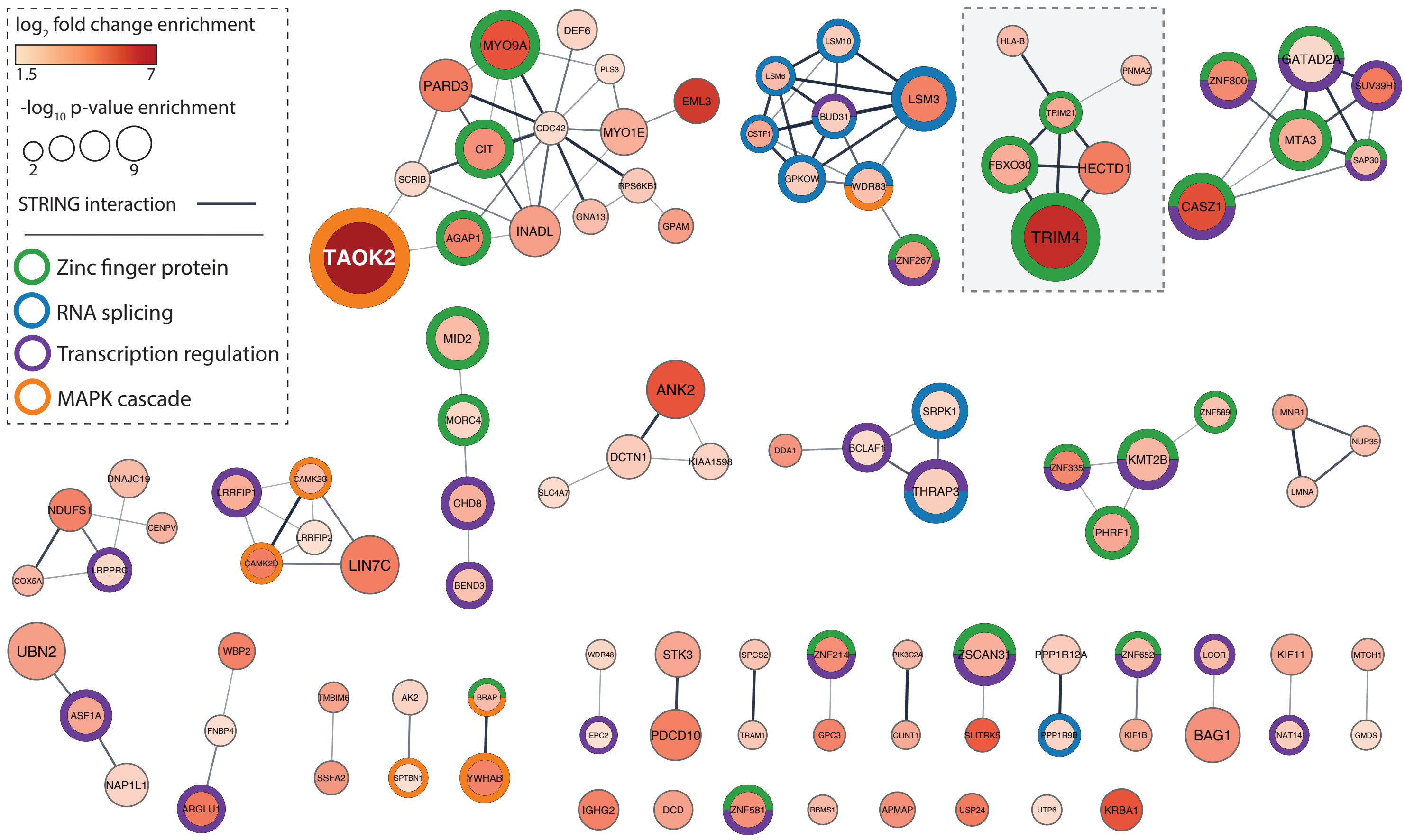

b

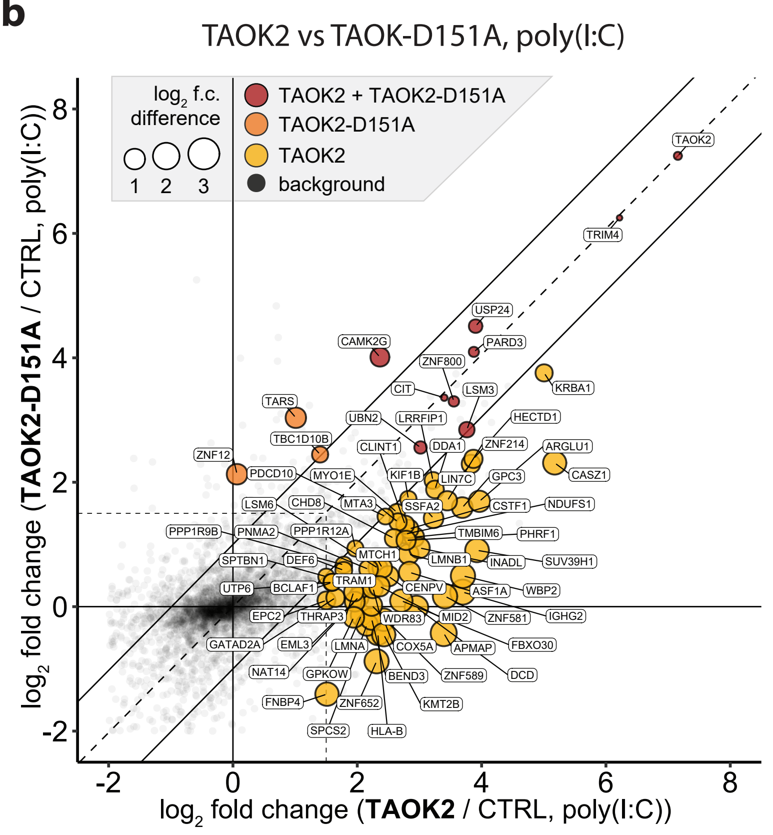

Supplementary Figure 9: TAOK2 AP-MS supplementary data

(a) STRING enriched network of rat TAOK2 interacting proteins in mock and poly(I:C) stimulated cells. Significantly enriched proteins were identified by a two-sided Student's T-tests (permutation-based FDR < 0.05) and further filtered to show a $\log_2$ fold change of ≥ 1.5. Proteins are colored and sized according to their $\log_2$ fold change enrichment or $-\log_{10}$ p-value in TAOK2 versus control comparisons, respectively. A confidence cutoff of 0.2 was set to filter for functional connections and an MCL inflation parameter of 4 was used to cluster the STRING-enriched network. Zinc finger proteins are highlighted in green, proteins regulating RNA splicing in blue, proteins involved in transcription regulation in purple and proteins of the MAPK cascade in orange. **(b)** Scatter plot comparing the $\log_2$ fold change enrichment of proteins following affinity purification of wild-type rat TAOK2 (*x*-axis) versus rat TAOK2-D151A (*y*-axis) in poly(I:C) stimulated HEK293T cells. Significantly enriched proteins were identified by a two-sided Student's T-tests (permutation-based FDR < 0.05), further filtered to show a $\log_2$ fold change of ≥ 1.5, and colored in yellow (only significant in wild-type TAOK2 with a $\log_2$ fold change difference ≥ 1 between wild-type and D151A-mutated TAOK2 affinity purifications), orange (only significant in TAOK2-D151A with a $\log_2$ fold change difference ≥ 1 between D151A-mutated and wild-type TAOK2 affinity purifications), red (significant in wild-type and D151A-mutated TAOK2) or black (non-significant, significant but a $\log_2$ fold change < 1.5, or significant and a $\log_2$ fold change ≥ 1.5 but a $\log_2$ fold change difference < 1). Point size corresponds to the absolute $\log_2$ fold change difference of a given protein between wild-type and D151A-mutated TAOK2 affinity purifications. Log_2 fold change difference values for each protein were normalized by the $\log_2$ fold change difference of TAOK2 to account for differences in enrichment efficiencies between the two TAOK2 variants.

Supplementary Table 1

Target	Primer-Sequence (forward)	Primer-Sequence (reverse)
GAPDH	GATTCCACCCATGGCAAATTC	AGCATCGCCCCACTTGATT
SFV	GCAAGAGGCAAACGAACAGA	GGGAAAAGATGAGCAAACCA
MX1	TGGAGGCACTGTCAGGAGTT	CCACAGCCACTCTGGTTATG
CrPV	GCTGAAACGTTCAACGCATA	CCACTTGCTCCATTTGGTTT
DCV	TCATCGGTATGCACATTGCT	CGCATAACCATGCTCTTCTG
FHV	TTTAGAGCACATGCGTCCAG	CGCTCACTTTCTTCGGGTTA
SINV	CAAATGTGCCACAGATACCG	ATACCCTGCCCTTTCAACAA
VSV	CATGATCCTGCTCTTCGTCA	TGCAAGCCCGGTATCTTATC
srg-1	GTGTCCATTATCCGCACAAG	ACTGGGGTATCTGACGGATG
diedel	GAGGAGGAACCAGCAGTACG	GGTTAAAATGGCAGCCTGGT
Rp49	GCCGCTTCAAGGGACAGTATCT	AAACGCGGTTCTGCATGAG

Supplementary Table 1: qPCR primer sequences
