# Supplementary material for: Cross-species analysis of viral nucleic acid interacting proteins identifies TAOKs as innate immune regulators
Source: Nat Commun. 2021 Dec 1;12:7009. doi: 10.1038/s41467-021-27192-w (PMC8636641; doi:10.1038/s41467-021-27192-w)
Supplement: Supplementary file 3 — Description of Additional Supplementary Files [file 41467_2021_27192_MOESM3_ESM.pdf]

Description of Additional Supplementary Files

File Name: Supplementary Data 1
Description: **Overview of Baits used.** Table detailing the different baits and controls used throughout the screening.

File Name: Supplementary Data 2
Description: **THP-1 AP-MS results.** AP-MS analysis of 17 NA affinity purifications in human THP-1 cells.

File Name: Supplementary Data 3
Description: **RAW AP-MS results.** AP-MS analysis of 17 NA affinity purifications in mouse RAW cells.

File Name: Supplementary Data 4
Description: **Fly AP-MS results.** AP-MS analysis of 15 NA affinity purifications in lysate from whole flies.

File Name: Supplementary Data 5
Description: **S2 AP-MS results.** AP-MS analysis of 12 NA affinity purifications in fly S2 cells.

File Name: Supplementary Data 6
Description: **Overlap between the human THP-1 AP-MS data and the SINV dataset and Reactome pathway analysis.** Overlap between the human THP-1 AP-MS data set and the poly-A RNA interactome in Sindbis virus infected cells as reported by Garcia et al. [17] and cellular host and restriction factors as reported by Tripathi et al. [22]. As well as the full results of the Reactome pathway analysis of all significantly enriched NA interactors independent of bait.

File Name: Supplementary Data 7
Description: **Domain Enrichment.** Domain enrichment performed for the 904 human AP-MS interactors.

File Name: Supplementary Data 8
Description: **Orthologue analysis – Mouse.** Orthologue analysis for the mouse NA interactors identified by AP-MS, sorted per bait.

File Name: Supplementary Data 9
Description: **Orthologue analysis – Fly and S2.** Orthologue analysis for the combined NA interactors identified by AP-MS of the whole fly lysate and the S2 cell lysate, sorted per bait.

File Name: Supplementary Data 10
Description: **sgRNA sequences and KD fly lines**. sgRNA sequences targeting 90 candidate genes and the positive control STAT1 selected using the GPP sgRNA designer. KK and GD inverted repeat transgenic fly lines from VDRC stock center used to induce the knockdown of candidate genes.

File Name: Supplementary Data 11
Description: **Full proteome analysis of TAOK KO cells.** Full proteome analysis of the three different TAOK-KO THP-1 cell lines including controls in mock and SFV infected conditions.

File Name: Supplementary Data 12
Description: **Affinity purification mass spectrometry analysis of TAOK2.** Affinity purification mass spectrometry analysis of wild-type rat TAOK2, rat TAOK2-R702C and rat TAOK2-D151A in mock or poly(I:C) stimulated HEK293T cells.
