# Supplementary material for: Cross-species analysis of viral nucleic acid interacting proteins identifies TAOKs as innate immune regulators
Source: Nat Commun. 2021 Dec 1;12:7009. doi: 10.1038/s41467-021-27192-w (PMC8636641; doi:10.1038/s41467-021-27192-w)
Supplement: Supplementary file 16 — Reporting Summary [file 41467_2021_27192_MOESM16_ESM.pdf]

nature portfolio

Corresponding author(s): Andreas Pichlmair

Last updated by author(s): Sep 24, 2021

Reporting Summary

Nature Portfolio wishes to improve the reproducibility of the work that we publish. This form provides structure for consistency and transparency in reporting. For further information on Nature Portfolio policies, see our Editorial Policies and the Editorial Policy Checklist.

Statistics

For all statistical analyses, confirm that the following items are present in the figure legend, table legend, main text, or Methods section.

n/a	Confirmed	
☐	☒	The exact sample size (n) for each experimental group/condition, given as a discrete number and unit of measurement
☐	☒	A statement on whether measurements were taken from distinct samples or whether the same sample was measured repeatedly
☐	☒	The statistical test(s) used AND whether they are one- or two-sided<br>*Only common tests should be described solely by name; describe more complex techniques in the Methods section.*
☒	☐	A description of all covariates tested
☐	☒	A description of any assumptions or corrections, such as tests of normality and adjustment for multiple comparisons
☐	☒	A full description of the statistical parameters including central tendency (e.g. means) or other basic estimates (e.g. regression coefficient) AND variation (e.g. standard deviation) or associated estimates of uncertainty (e.g. confidence intervals)
☐	☒	For null hypothesis testing, the test statistic (e.g. F, t, r) with confidence intervals, effect sizes, degrees of freedom and P value noted<br>*Give P values as exact values whenever suitable.*
☐	☒	For Bayesian analysis, information on the choice of priors and Markov chain Monte Carlo settings
☒	☐	For hierarchical and complex designs, identification of the appropriate level for tests and full reporting of outcomes
☒	☐	Estimates of effect sizes (e.g. Cohen's d, Pearson's r), indicating how they were calculated

Our web collection on statistics for biologists contains articles on many of the points above.

Software and code

Policy information about availability of computer code

Data collection	IncuCyte Analysis Software (2019B Rev2), Maxquant (1.5.0.0, 1.6.14.0, 1.6.17.0), QuantStudio Design and Analysis Software (1.4.3)
Data analysis	GraphPad Prism (9.1.0), IncuCyte Analysis Software (2019B Rev2), Maxquant (1.5.0.0, 1.6.14.0, 1.6.17.0), Perseus (1.5.2.1, 1.6.13.0, 1.6.15.0), R (3.5.0, 4.1.0), R Studio (1.4.1717), Julia(1.5), Cytoscape (3.8.2) + stringApp (1.6.0), Gene Set Enrichment Analysis: OptEnrichedSetCover.jl (0.5.0, https://github.com/alyst/OptEnrichedSetCover.jl), Affinity Analysis (2.2.4, NanoTemper Technologies MO)

For manuscripts utilizing custom algorithms or software that are central to the research but not yet described in published literature, software must be made available to editors and reviewers. We strongly encourage code deposition in a community repository (e.g. GitHub). See the Nature Portfolio guidelines for submitting code & software for further information.

Data

Policy information about availability of data

All manuscripts must include a data availability statement. This statement should provide the following information, where applicable:

- Accession codes, unique identifiers, or web links for publicly available datasets
- A description of any restrictions on data availability
- For clinical datasets or third party data, please ensure that the statement adheres to our policy

The mass spectrometry proteomics data have been deposited to the ProteomeXchange Consortium (http://proteomecentral.proteomexchange.org) via the PRIDE partner repository with the dataset identifier PXD027894, PXD027896 and PXD027919.

Field-specific reporting

Please select the one below that is the best fit for your research. If you are not sure, read the appropriate sections before making your selection.

☒ Life sciences ☐ Behavioural & social sciences ☐ Ecological, evolutionary & environmental sciences

For a reference copy of the document with all sections, see nature.com/documents/nr-reporting-summary-flat.pdf

Life sciences study design

All studies must disclose on these points even when the disclosure is negative.

Sample size	All cell-line based experiments and in vitro assays have been performed at least three times. Sample sizes were chosen from past knowledge to ensure adequate statistical power. Sample sizes are always indicated in figure legends or the related Methods section.
Data exclusions	Data was not excluded.
Replication	If not stated otherwise three biological experiments were performed and all attempts were successful at replication.
Randomization	N/A. No randomization was used given the small number of samples and the lack of influence of randomization on the experimental design and experimental approach used.
Blinding	N/A. Investigators were not blinded to experimental groups since in vitro experiments required prior knowledge for data interpretation.

Reporting for specific materials, systems and methods

We require information from authors about some types of materials, experimental systems and methods used in many studies. Here, indicate whether each material, system or method listed is relevant to your study. If you are not sure if a list item applies to your research, read the appropriate section before selecting a response.

Materials & experimental systems

n/a	Involved in the study	
☐	☒	Antibodies
☐	☒	Eukaryotic cell lines
☒	☐	Palaeontology and archaeology
☐	☒	Animals and other organisms
☒	☐	Human research participants
☒	☐	Clinical data
☒	☐	Dual use research of concern

Methods

n/a	Involved in the study	
☒	☐	ChIP-seq
☒	☐	Flow cytometry
☒	☐	MRI-based neuroimaging

Antibodies

Antibodies used	α-β-Actin-HRP (Santa Cruz: sc-47778), α-TAOK1 (Bethyl Laboratories: A300-524A-M), α-TAOK2 (Sigma-Aldrich: HPA010650), α-TAOK3 (Sigma-Aldrich: HPA017160), α-ABCF1 (Aviva Sytems Biology: ARP43631_P050), α-ABCF3 (Sigma: HPA036332), α-RNASEL (gift from Robert Silverman, Lerner Research Institute, Ohio, USA), α-RSL1D1 (Sigma: HPA043483), α-SFV core (gift from Andres Mertis, University of Tartu, Estonia), α-SMARCA5 (Sigma: HPA008751), α-STAT1 (Cell Signaling Technology: 9172), α-ms-IgG-HRP (Sigma-Aldrich: A0168), α-rb-IgG-HRP (Cell Signaling Technology: 7074), α-MX1 (gift from Georg Koch, University of Freiburg, Germany), α-PARP12 (Sigma: HPA063872)
Validation	All antibodies were validated by the company or in-house using knockout or overexpression cell lines.

Eukaryotic cell lines

Policy information about cell lines

Cell line source(s)	HEK293T (ATCC, CRL-3216), THP-1 (gift from Veit Hornung, Gene Center Munich, Germany), A549 (gift from Georg Kochs, University of Freiburg, Germany), A549-IFIT1-eGFP (gift from Ralf Bartenschlager, Heidelberg University, Germany), RAW263.7 (gift from Thomas Decker, MFPL Vienna, Austria), Schneider S2 (gift from Irene Ferreira)
Authentication	Cell lines were authenticated by the company and validated by STR profiling (Eurofins Medigenomix).
Mycoplasma contamination	Cell lines were tested negative for mycoplasma contamination by a standard PCR-based assay.

Commonly misidentified lines (See ICLAC register)	No commonly misidentifed cell lines were used, all cell-lines were authenticated by ATCC.

Animals and other organisms

Policy information about studies involving animals; ARRIVE guidelines recommended for reporting animal research

Laboratory animals	Drosophila melanogaster, male and female, 6-8 days old adult flies
Wild animals	No wild animals were used.
Field-collected samples	No field-collected samples were used.
Ethics oversight	No ethical approvement was required.

Note that full information on the approval of the study protocol must also be provided in the manuscript.
