# Supplementary material for: Cross-species analysis of viral nucleic acid interacting proteins identifies TAOKs as innate immune regulators
Source: Nat Commun. 2021 Dec 1;12:7009. doi: 10.1038/s41467-021-27192-w (PMC8636641; doi:10.1038/s41467-021-27192-w)

S1b SMARCA5 Blot

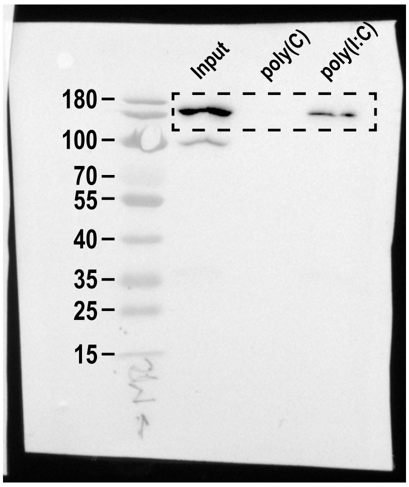

S1b SMARCA5 β-actin Blot

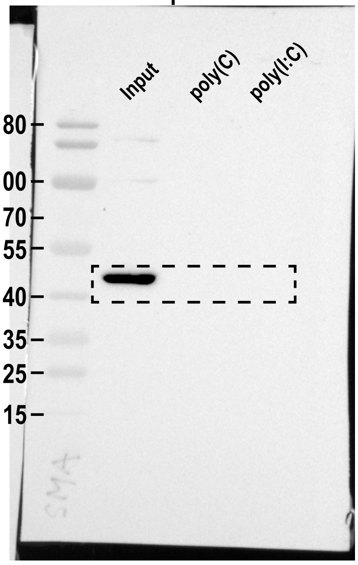

S1b PARP12 Blot

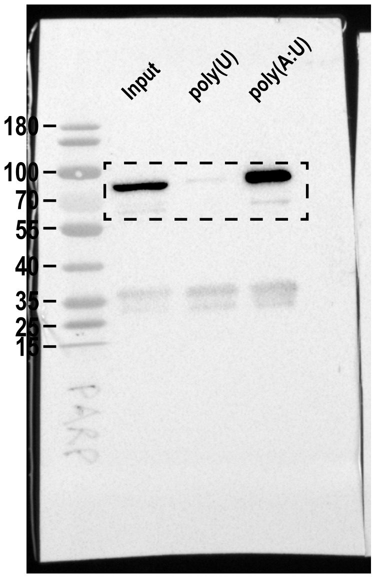

S1b PARP12 β-actin Blot

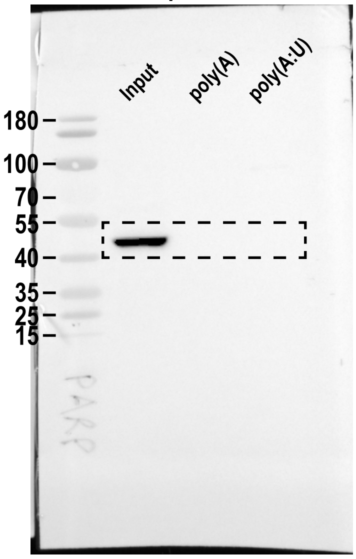

S1b ABCF1 Blot

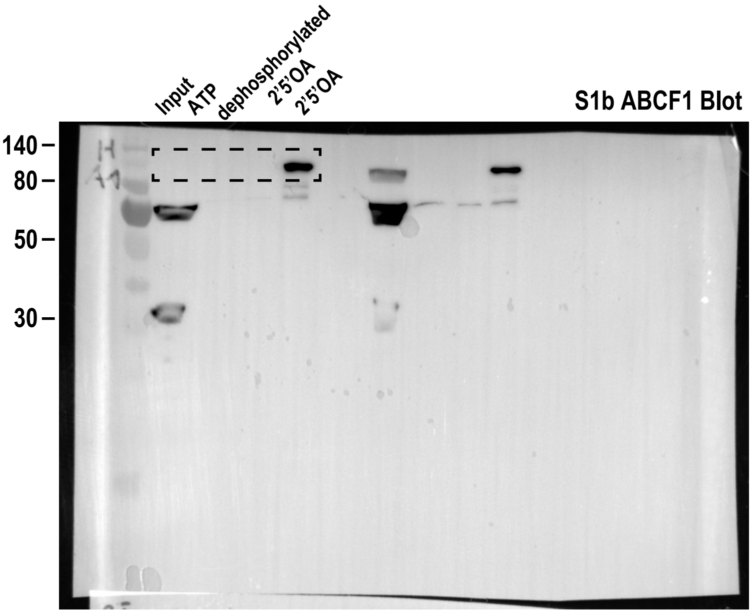

S1b ABCF1 RNAseL Blot

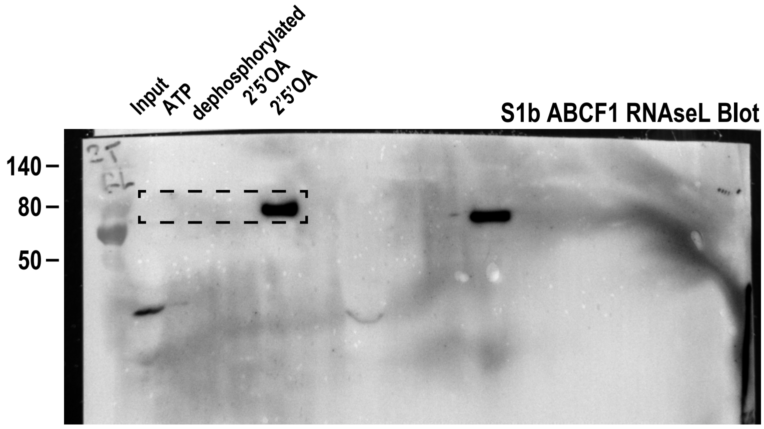

S1b ABCF3 Blot

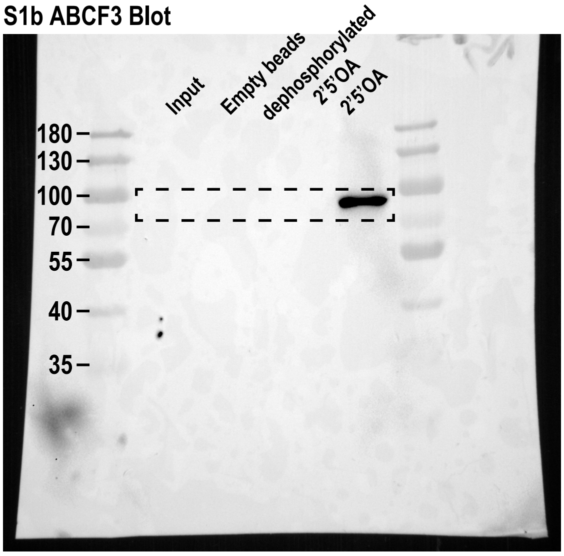

S1b ABCF3 RNaseL Blot

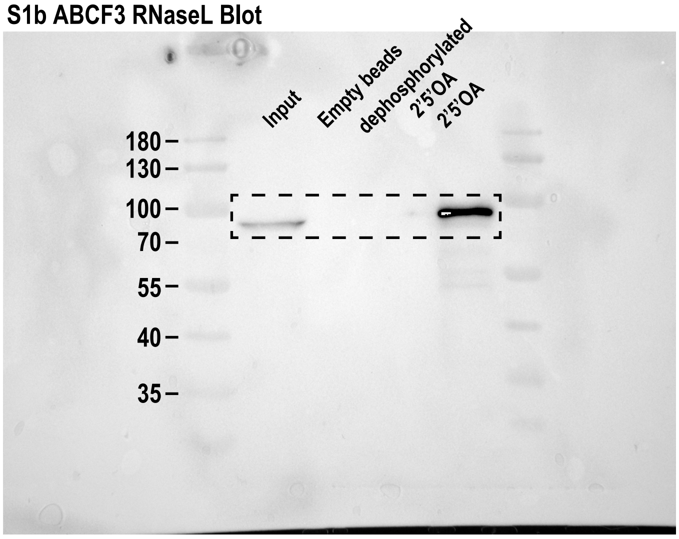

Supplement: Supplementary file 17 — Source Data [file 41467_2021_27192_MOESM17_ESM.zip › Supplementary Figure 1b.pdf]
