# Supplementary figures and images for: Cross-species analysis of viral nucleic acid interacting proteins identifies TAOKs as innate immune regulators
Source: Nat Commun. 2021 Dec 1;12:7009. doi: 10.1038/s41467-021-27192-w (PMC8636641; doi:10.1038/s41467-021-27192-w)

5a TAOK1 Blot

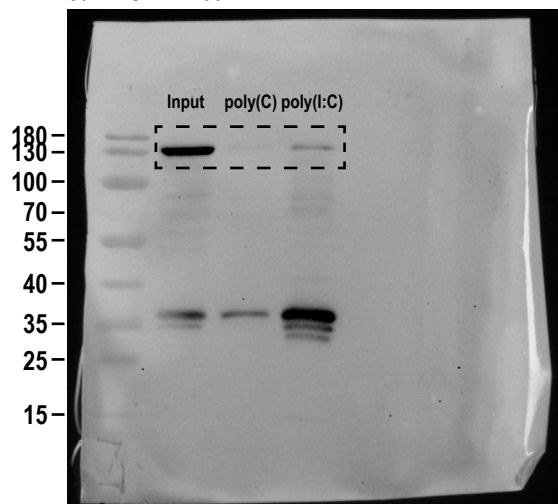

5a TAOK2 Blot

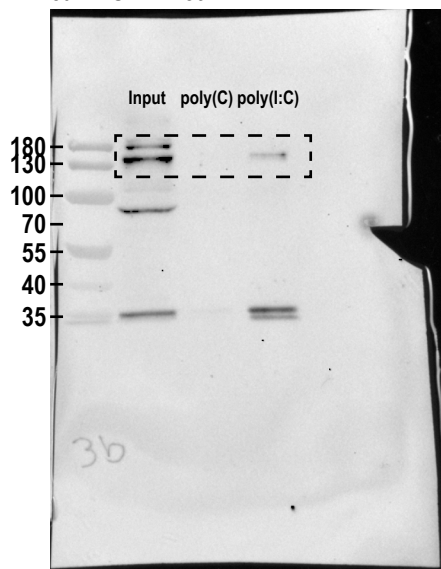

5a TAOK3 Blot

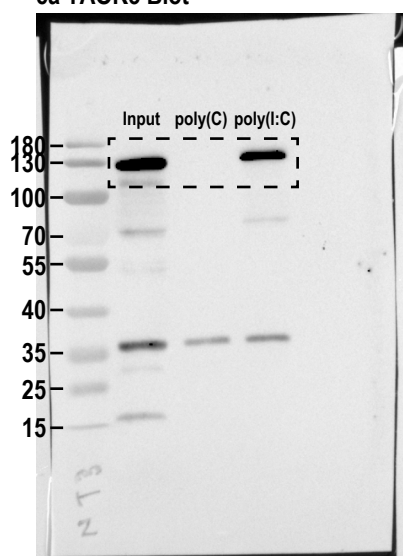

5a  $\beta$ -actin Blot

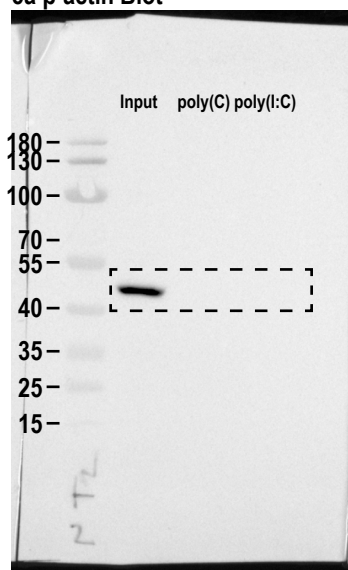

Supplement: Supplementary file 17 — Source Data [file 41467_2021_27192_MOESM17_ESM.zip › Figure 5a.pdf]

6b Mock/SFV MX1 Blot

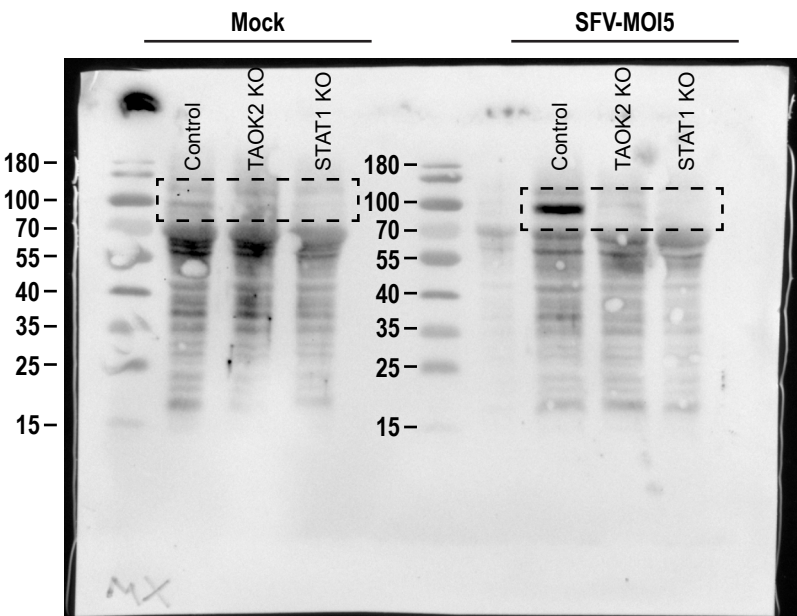

6b Mock/SFV  $\beta$ -actin Blot

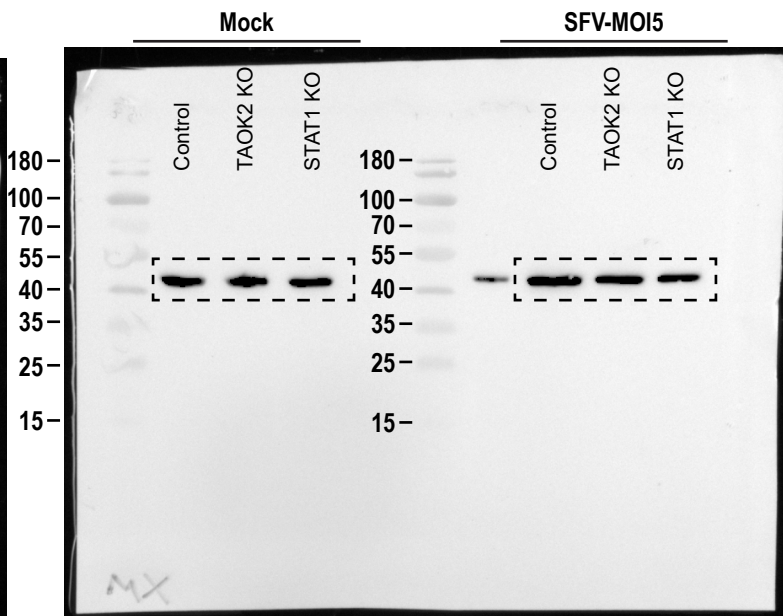

6b Mock/SFV SFV Blot

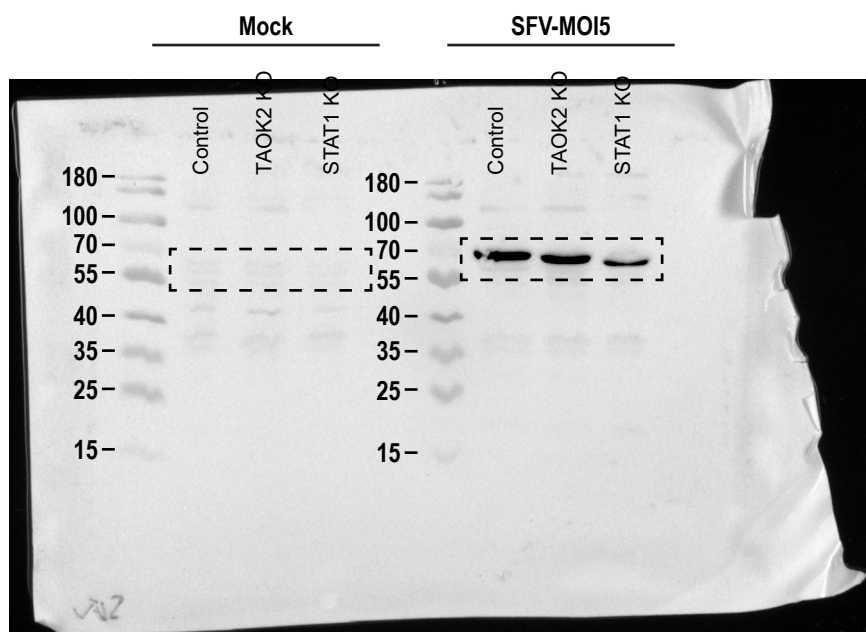

6b IFN MX1 Blot

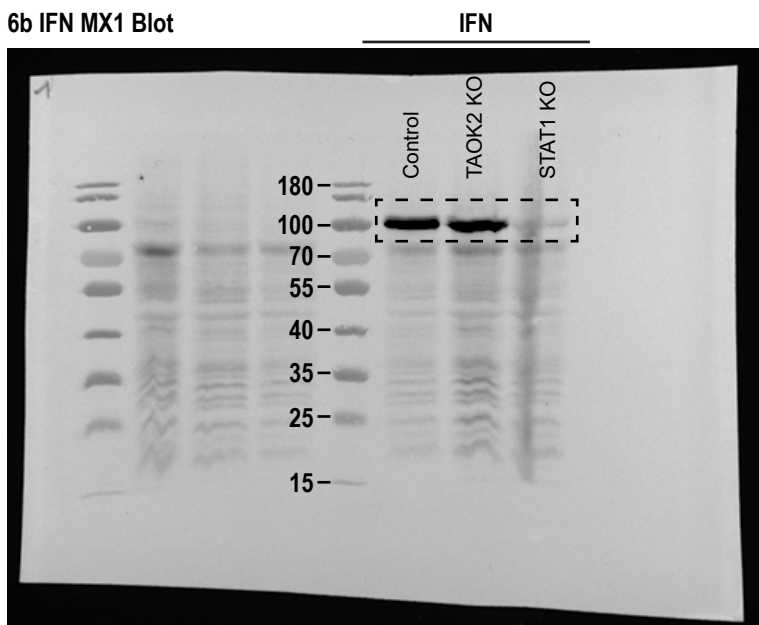

6b IFN  $\beta$ -actin Blot

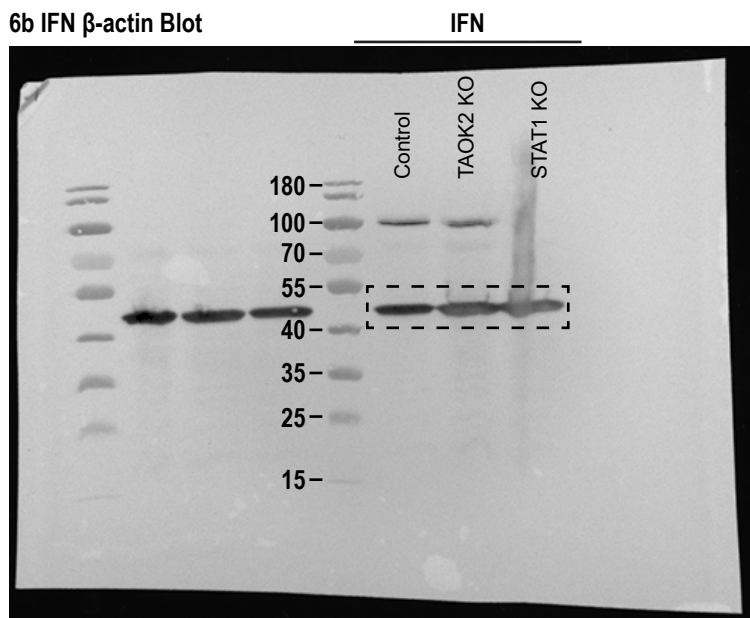

Supplement: Supplementary file 17 — Source Data [file 41467_2021_27192_MOESM17_ESM.zip › Figure 6b.pdf]

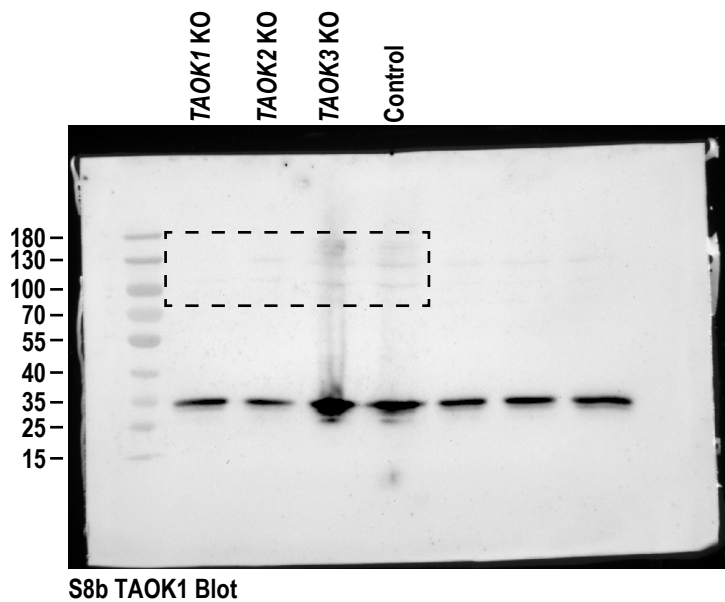

S8b TAOK1 Blot

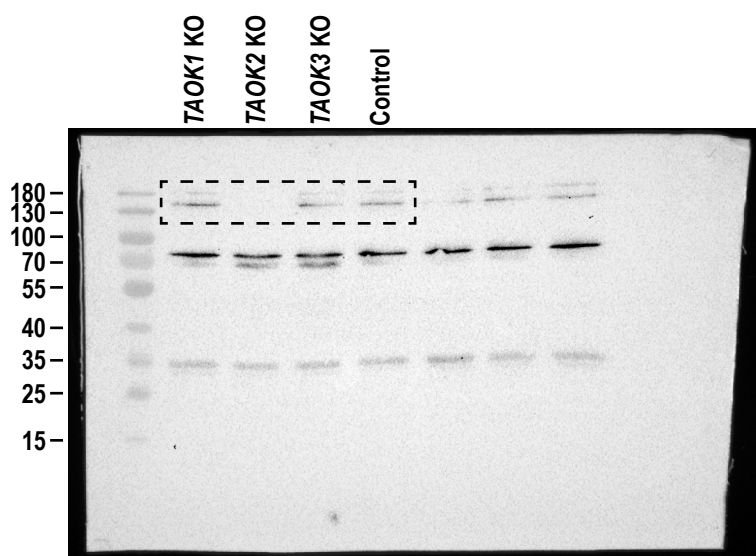

S8b TAOK2 Blot

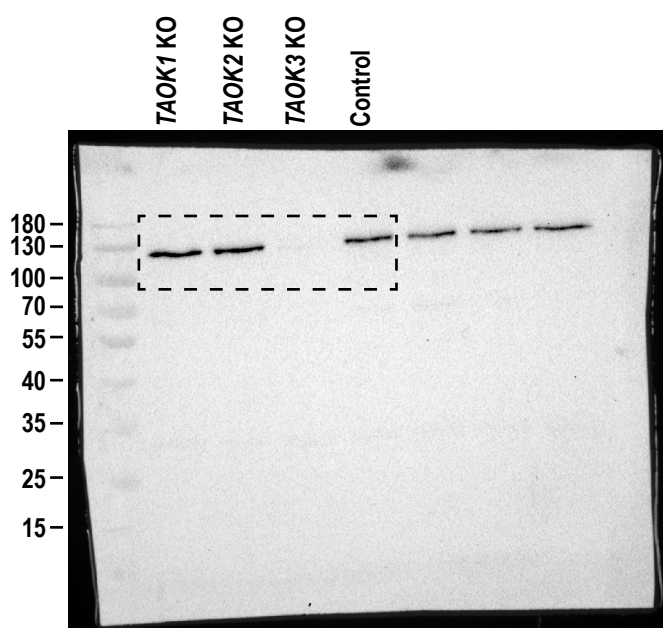

S8b TAOK3 Blot

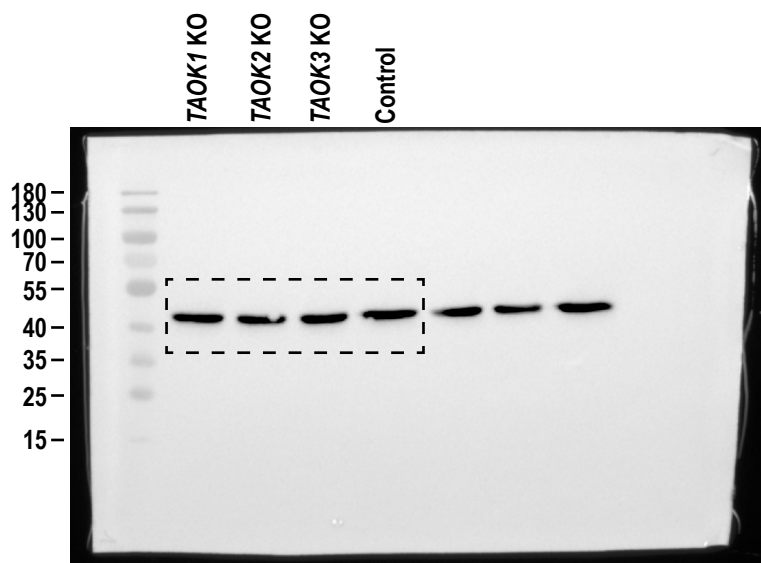

S8b β-actin Blot

Supplement: Supplementary file 17 — Source Data [file 41467_2021_27192_MOESM17_ESM.zip › Supplementary Figure 8b.pdf]

S8c TAOK2 Blot

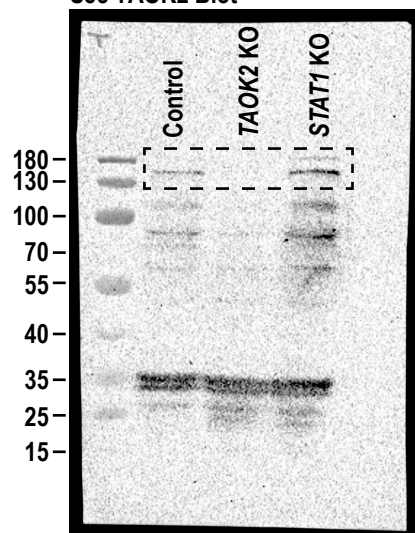

S8c STAT1 Blot

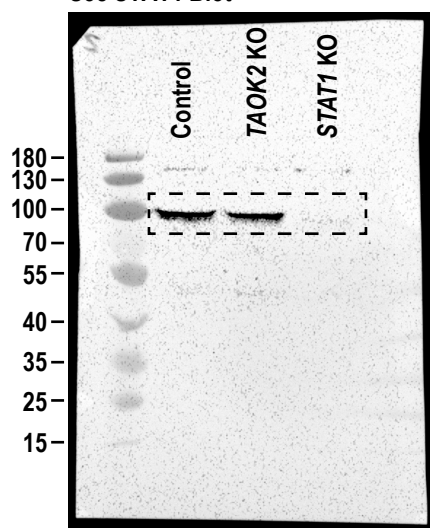

S8c  $\beta$ -actin Blot

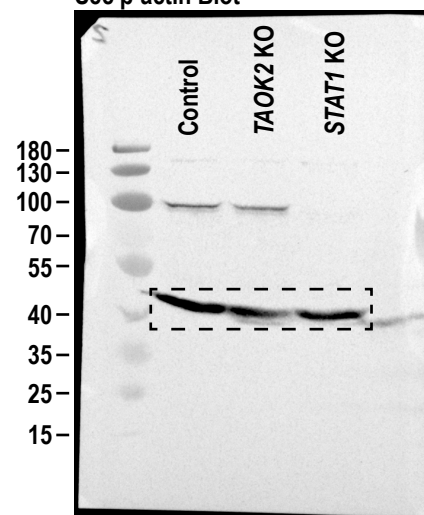

Supplement: Supplementary file 17 — Source Data [file 41467_2021_27192_MOESM17_ESM.zip › Supplementary Figure 8c.pdf]
